# The Role of Attachment in Emotional Support Provision in Adult Child–Parent Relationships: A Dyadic Response Surface Analysis

**DOI:** 10.3390/bs16010106

**Published:** 2026-01-13

**Authors:** Ella Carasso, Dikla Segel-Karpas, Roi Estlein

**Affiliations:** 1 Department of Gerontology, University of Haifa, Haifa 3498838, Israel; 2 School of Social Work, University of Haifa, Haifa 3498838, Israel

**Keywords:** attachment theory, emotional support, interpersonal processes

## Abstract

The adult child–parent relationship is a key source of emotional support across adulthood and older age. This study takes a dyadic, attachment-based perspective to examine how (dis)similarities in attachment orientations between older parents and adult children relate to the emotional support they provide each other. A total of 104 adult child–parent dyads (M parents’ age = 67.85; M adult children’s age = 36.18) participated. Structural Equation Modeling (SEM) and Response Surface Analysis (RSA) were used to assess how dyadic (dis)similarities in attachment anxiety and avoidance are associated with own support provision. Both parents and adult children provided greater emotional support when their attachment insecurity was at low levels. Support also increased when the partner showed higher insecurity but differed across generations: parents offered more support when the child’s anxiety or avoidance exceeded their own, even at own high levels of insecurity, whereas children supported insecure parents only when their own insecurity was relatively low. Attachment-based processes in the adult child–parent bond serve as a source of emotional connection, operating differently across generations: parents can adapt caregiving to meet children’s needs, while children’s support is more constrained by their own attachment insecurity.

## 1. Introduction

Throughout adulthood and later life, the adult child–parent relationship is considered an essential foundation for mutual emotional support (Fingerman & Birditt, 2011; Fingerman et al., 2016; Morais et al., 2024). Expressed through care, empathy, and understanding, emotional support is considered a significant indicator of family cohesion, helping both generations navigate major life transitions (Bui et al., 2022; Damman & van Duijn, 2017; Min et al., 2022). While aging parents often face the experience of loss associated with retirement, an empty nest, and the passing of loved ones, adult children are typically focused on establishing independence and developing marital and familial relationships (Boerner et al., 2022; Carter & McGoldrick, 2005). These contrasting life transitions can create tension and differing emotional needs, with warmth and emotional support playing an essential role in promoting social connectedness between adult children and their parents and are associated with better well-being for both generations (Carr & Utz, 2020; Jessee & Carr, 2025).

Intergenerational relationships between older parents and adult children have been widely examined through sociological frameworks, investigating the significant role of shared familial and cultural values, and norms of filial obligation in maintaining supportive ties (Charenkova, 2023; Fingerman & Birditt, 2011; Fingerman et al., 2012; Min et al., 2012). Our study seeks to contribute to the efforts in explaining intergenerational support by offering a psychological perspective, examining how individual factors can shape the extent to which adult child–parent relationship serves as an emotional support resource for both generations.

We draw on attachment theory (Allen, 2023; Bowlby, 1969/1982), which was originally developed to explain early child–parent relationships based on emotional connection and supportive caregiving in early life and later extended to understanding the role of supportive attachment bond in adulthood, including relationships between aging parents and their adult children (Cicirelli, 2010; Mikulincer & Shaver, 2023b). Attachment theory offers a framework for understanding how individual differences in behavioral motivations in relationships contribute to support processes and maintenance of emotionally supportive ties (Collins & Feeney, 2000, 2004; Mikulincer & Shaver, 2023a, 2023b).

A central concept in the attachment theory is that internal schemas, known as working models, shape individuals’ inclination to seek and feel comfortable with relational support while also guiding their caregiving behaviors, designed to alleviate partner’s distress, demonstrating responsiveness, and offering support (Mikulincer & Shaver, 2003, 2023a, 2023b). Based on the theory, the literature on adult attachment describes support provision as a reciprocal process, shaped by one partner’s working models, which determine how they express their need for support, and the other partner’s models, which guide how they perceive and respond to these needs (Collins & Feeney, 2000; Chin & Feeney, 2022).

This study employs a dyadic approach to examine how attachment functions as an internal mechanism shaping the motivation of adult children and their parents to exchange emotional support. We aim to explore how differences and similarities in the attachment models of older parents and adult children shape the extent to which they provide emotional support to one another. Understanding these potential (dis)similarities can help explain how the emotional support older parents and children offer each other may vary based on the reciprocal functioning of their attachment models. This approach allows for exploring the variations in relationship patterns, which could either expose the adult child–parent relationships to the risk of disconnection and strain or contribute to healthy and supportive bonds throughout adulthood and later life (B. C. Feeney & Collins, 2015; Jessee & Carr, 2025). With this, we can shed light on how attachment, which shapes interpersonal characteristics between children and parents in early life, plays a role in shaping emotional support as a key relational resource through the later stages of their relationships.

### 1.1. Attachment Theory

Attachment theory (Bowlby, 1969/1982; O’Shaughnessy et al., 2023) was developed to explain the formation of early child–parent bonds. According to the theory, individuals are born with an innate attachment system that, when activated, motivates them to seek closeness and support of their primary caregivers in times of distress and separation. The child’s ability to rely on their caregivers for support and protection promotes distress alleviation and formation of a sense of subjective security. The main premise of the theory is that individual’s attempts to attain felt security through repeated interactions with significant caregivers, promotes the development of internalized working models which incorporate individual’s perceptions of whether they deserve of love and support, and whether others can be trusted to be responsive and supportive of their needs (Collins & Feeney, 2000, 2004; Mikulincer & Shaver, 2023a, 2023b).

### 1.2. Adult Attachment and Support Processes

Bowlby (1969/1982) argued that attachment models continue to guide social behavior and emotional responses within interpersonal connections through adulthood. Research on adult attachment suggests that based on their attachment-related experiences, individuals vary in attachment models functioning (Mikulincer & Shaver, 2003, 2023a). These differences are thought to motivate not only support-seeking behaviors, as observed in early life, but also caregiving behaviors aimed at responding and meeting the emotional and psychological needs of others (Collins & Feeney, 2000, 2004; Florian et al., 2010; McLeod et al., 2020).

Two central dimensions are considered to underlie the operation of adult attachment models: attachment anxiety and attachment avoidance. The combination between these two dimensions creates different attachment orientations which reflect the operation of individuals’ dominant attachment models. The anxiety dimension represents the extent to which individuals view themselves as being worthy of others’ support and the extent to which they worry about being rejected. The avoidance dimension relates to the extent to which individuals perceive others as responsive and feel comfortable with intimacy (Bartholomew & Horowitz, 1991; Simpson et al., 2021; Mikulincer & Shaver, 2023b). Both dimensions shape individuals’ ability to perceive the availability of support and to provide responsive support to others.

Individuals who are low in attachment anxiety and avoidance are characterized by attachment security. They are confident in their self-value, feel comfortable relying on others for support and maintaining emotionally close relationships. A sense of security enables individuals to redirect their focus from the continual need for fulfilling personal needs, and promotes emotional availability for caregiving, showing more empathy and compassion and offering appropriate support (Mikulincer et al., 2001, 2005; Mikulincer & Shaver, 2020, 2023b).

Adults who are high in attachment anxiety tend to see themselves as unworthy of other’s care and fear rejection, often leading to an intensified need for reassurance and a strong desire for emotional intimacy. Their tendency to focus on their own unmet needs can interfere with their ability to provide attentive and effective care (Collins & Feeney, 2000, 2004; Mikulincer & Shaver, 2020, 2023b). Moreover, research suggests that while individuals high in anxiety are comfortable with the intimacy required for providing support, their ability to meet their partner’s needs may be limited as their motivations to support them are often driven by personal need for validation and fear of rejection and separation (Mikulincer & Shaver, 2003, 2023b).

Adults who are high in attachment avoidance often prioritize independence and self-reliance, viewing others as unreliable and preferring emotional distance. To preserve positive self-image, they tend to minimize their own need for other’s care and may see expressions of emotional reliance as unwanted (Mikulincer & Shaver, 2003, 2023a). Their preference for emotional distance also limits their ability to effectively support their partner, as they often respond with withdrawal and dismiss their partner’s needs in interactions that require emotional involvement (Collins & Feeney, 2004; Mikulincer & Shaver, 2020, 2023b).

Dyadic research on adult attachment has shown that the expression of support is part of an interpersonal process that involves the interaction between one partner’s attachment orientation in seeking support and the other partner’s attachment-based caregiving response (Collins & Feeney, 2000; J. A. Feeney & Hohaus, 2001; Mikulincer & Shaver, 2020, 2023b). Interindividual differences in attachment-related anxiety and avoidance reflect how individuals experience and respond to support and how they interpret partner’s needs (Mikulincer & Shaver, 2003, 2023a). Thus, support expression in relationships is shaped by each partner’s attachment orientation and these reciprocal effects at the dyadic level shape the extent to which the relationship serves as a framework of support. Specifically, it is the degree of similarity or difference between partners’ levels of attachment-related anxiety and avoidance that may generate support exchange dynamics.

### 1.3. Attachment Similarity and Support Provision

A significant body of research on adult attachment has focused on understanding support processes by investigating how attachment shapes support-seeking and caregiving behaviors in close relationships (e.g., Collins & Feeney, 2000; Florian et al., 2010; Mikulincer & Shaver, 2023a). However, there is limited evidence on how the interaction of differences and similarities in attachment orientations between relational partners generate support processes. Based on theoretical understanding, it can be assumed that while partners who share low levels of anxiety and avoidance may express more emotional responsiveness and support, these similarities at higher levels may have the opposite effect. Highly anxious dyads may focus on their own emotional distress, limiting their ability to address partner’s needs, while the highly avoidant dyads may be prone to emotional distance and poor emotional communication (Mikulincer & Shaver, 2003, 2023a, 2023b). As parents and adult children may not align in their attachment orientation, these interindividual differences when one partner is lower in anxiety and avoidance than the other, may allow them to provide more effective care and help them regulate their partner’s distress (Mikulincer & Shaver, 2023a, 2023b; Simpson & Overall, 2014). On the other hand, these differences can also occur when both partners experience attachment insecurity, with one being higher in anxiety or avoidance than the other. These dyads may struggle to offer effective support, as anxious partners tend to be preoccupied with their own unmet needs, while avoidant partners prioritize emotional distance (Mikulincer & Shaver, 2003, 2023a, 2023b). Research suggests that these dyads are particularly prone to demand-withdraw dynamics, where the highly anxious partner often actively seeks support while the avoidant partner feels overwhelmed by these constant emotional demands and minimizes emotional involvement, reflecting their conflicting needs for emotional connection (Conradi et al., 2021).

While the knowledge on support processes from an attachment perspective is largely rooted in research on dyads within romantic relationships (Fogel-Yaakobi et al., 2023; Simpson et al., 2002), there is less research on the dynamics of support between older parents and their adult children. Prior research on intergenerational relationships has indicated the importance of examining adult child–parent relationships from an attachment perspective, as attachment processes are central to family functioning and may shape caregiving in later stages of the relationship, when support exchanges between parents and children become increasingly bidirectional (Brandão et al., 2022; Merz et al., 2007). Additional work has emphasized the role of attachment processes in family functioning and has informed attachment-based therapeutic approaches aimed at understanding and improving family relationships (George & Wargo Aikins, 2023). Research on the role of attachment in family relationships, conducted within caregiving context, has demonstrated that attachment insecurity among caregivers of aging family members is associated with greater caregiver stress and negative affect (Wuestefeld et al., 2022). Furthermore, evidence indicates that interactions between adult children’s and parents’ attachment orientations are linked to caregivers’ subjective burden, underscoring the importance of considering attachment processes within a dyadic framework (Romano et al., 2020). To the best of our knowledge, studies examining bidirectional support processes outside contexts of dependency and caregiving in adult child-older parent relationships from a dyadic attachment perspective remain limited. The present study addresses this gap in the literature by employing a dyadic, attachment-based approach to investigate adult child–parent support relationships in adulthood and older age. Given that Intergenerational support is a central component of the child–parent bond in adulthood and later life and is associated with mental well-being and healthy aging (Bertogg & Manzoni, 2025; Fingerman & Birditt, 2011), examining attachment processes can clarify how parents and adult children maintain relationships that serve as sources of mutual support. This dyadic attachment-based perspective is particularly important across the life course and family development, as family roles, relational expectations, and patterns of support provision evolve from primarily hierarchical, parent-to-child caregiving in earlier life to more interdependent exchanges across adulthood and older age, while parents and adult children navigate distinct developmental tasks, including parents’ adjustment to aging and adult children’s pursuit of autonomy, underscoring the value of a dyadic perspective (Fingerman et al., 2020, 2024).

Drawing on attachment theory (Bowlby, 1969/1982, Mikulincer & Shaver, 2003, 2023a, 2023b), the present study aims to contribute to existing research in two key aspects. First, the study employs a dyadic approach to broaden the understanding of how attachment shapes emotional support exchanges between aging parents and adult children. Second, the study examines how attachment orientations interact to shape support processes, with a focus on how similarities and differences in attachment anxiety and avoidance between parents and adult children shape their mutual provision of emotional support. Through this investigation, we aim to deepen the understanding of how these attachment (dis)similarities relate to variations in emotional support provision within adult child–parent dyads.

Similarity in attachment orientation refers to situations in which parents and adult children report comparable levels of attachment anxiety or avoidance, whereas dissimilarity reflects discrepancies between partners’ attachment orientations (Song et al., 2024). Similarity may occur at either low or high levels of attachment insecurity, with potentially distinct implications: similarity at low levels of insecurity may facilitate mutual responsiveness and emotional availability, similarity at high levels may reflect shared distress or emotional distancing that could constrain support provision, and directional incongruence in parents’ and adult children’s levels of attachment anxiety and avoidance captures whether the parent’s attachment insecurity exceeds that of the adult child, or vice versa (Mikulincer & Shaver, 2003, 2023b). This distinction can be theoretically meaningful in adult child–parent relationships, to better understand how elevated attachment insecurity in one partner may differentially activate caregiving responses or place greater emotional demands on the other partner (Mikulincer & Shaver, 2023a, 2023b).

Theoretically, attachment anxiety and avoidance reflect distinct regulatory strategies for maintaining emotionally supportive connections (Mikulincer & Shaver, 2024). Anxiety is characterized by heightened distress signaling and proximity seeking, whereas attachment avoidance involves emotional distancing and the downregulation of attachment needs (Mikulincer & Shaver, 2023a, 2023b). We use Response Surface Analysis (RSA) (Schönbrodt, 2016; Song et al., 2024) to model parents’ and adult children’s attachment orientations within dyads, allowing us to examine how similarity, dissimilarity, and directional incongruence operate within comparable attachment processes. This approach enables meaningful comparisons within the same attachment dimension, clarifying how varying levels of anxiety or avoidance in adult child–parent dyads relate to emotional support provision.

We explore the following questions:

RQ1: How are (dis)similarities in levels of attachment anxiety between adult children and parents associated with the emotional support they provide to each other?

RQ2: How are (dis)similarities in levels of attachment avoidance between adult children and parents associated with the emotional support they provide to each other?

## 2. Materials and Methods

### 2.1. Procedure and Participants

A total of 104 adult child–parent dyads were recruited through research assistants and social media research groups in Israel. After contacting the researcher, participants were informed about the study’s objectives, target population, and had the opportunity to ask questions. They were informed that they could choose which parent, or child would participate with them. If both the parent and child consented after this discussion, a date was scheduled for them to complete the questionnaire. Following informed consent, each participant independently completed a questionnaire designed for either a parent or an adult child. Participants unable to provide informed consent for participation in the study due to medical or cognitive condition were not included. To ensure pairing of parents and children during data analysis, participants were asked to include the last five digits of both their own and their partner’s phone numbers in the questionnaire. This information was collected solely for the purpose of identifying dyads for data analysis and explained to the participants. The research was approved by the Institutional Review Board of the University of Haifa.

Participating parents’ age ranged from 54 to 89 (*M* = 67.7, *SD* = 7.35), with 95 (90.48%) being 60 years of age or older. Participating adult children’s age ranged from 22 to 56 (*M* = 36.18, *SD* = 8.71). The sample included 56 mother-daughter dyads, 25 father-daughter dyads, 11 father-son dyads, and 12 mother-son dyads. Of the total sample, 180 participants (86.54%) were Jewish, 12 (5.77%) were Muslim, 15 (7.21%) were Christians, and one participant (0.48%) did not report their religion. In terms of marital status, 72 (69.23%) of the parents were married, 14 (13.46%) divorced, 16 (15.38%) widowed, unmarried 1 (0.96%) and 1 (0.96%) reported “other”. Among the adult children, 49 (47.11%) were unmarried, 46 (44.23%) married, 6 (5.77%) divorced, 1 (0.96%) widowed, and 2 (1.92%) reported “other”. For parents, the mean number of children was 3.30, and for adult children it was 1.20.

### 2.2. Measures

#### 2.2.1. Emotional Support Provision

The 2-Way Social Support Scale (2-Way SSS) was used to evaluate provision of emotional support between adult children and their parents (Shakespeare-Finch & Obst, 2011). Each item was rated on a scale ranging from 1 (“strongly disagree”) to 5 (“strongly agree”). Items were: (a) I am always available to listen to my parent/child; (b) I try to encourage my parent/child when he/she is feeling sad or upset; (c) my parent/child feels comfortable sharing their fears and concerns with me; (d) I provide a sense of security for my parent/child when they are in need; (e) my parent/child knows they can rely on me (*α* = 0.89 for parents and *α* = 0.87 for adult children).

#### 2.2.2. Attachment Orientation

The ECR-R (Experiences in Close Relationships—Revised) (Fraley et al., 2000; Mikulincer & Shaver, 2023b) was used to explore how children and parents perceive support and intimacy within their relationship. Attachment orientation of adult children and their parents were assessed on two dimensions: attachment anxiety and attachment avoidance. Each item was rated on a scale ranging from 1 (“strongly disagree”) to 7 (“strongly agree”). (Attachment anxiety: *α* = 0.93 for parents and *α* = 0.94 for adult children; Attachment avoidance: *α* = 0.94 for parents and *α* = 0.95 for adult children).

### 2.3. Analytical Strategy

The data was organized such that each observation corresponded to an adult child–parent dyad. Separate models were constructed to examine how (dis)similarities in attachment orientations between parents and adult children are associated with their own emotional support provision. Data were analyzed using R (v.4.3.2) with the lavaan package (v.0.6-16) (Rosseel, 2012) and the RSA package (v.0.10.6) (Schönbrodt & Humberg, 2023) in RStudio (v.2023.09.1). The independent variables were centered prior to the analysis.

The present study employs Response Surface Analysis (RSA), an analytic approach that allows for the examination of how similarity and dissimilarity between two predictor variables are associated with outcome variable (Schönbrodt, 2016). Using this analytical approach, enabled the investigation of how (dis)similarity in attachment anxiety and avoidance are related to higher or lower levels of own emotional support provision.

To date, RSA has been widely applied to the study of child–parent relationships in adolescence (Hu & Zhou, 2024; Song et al., 2024), with emerging evidence suggesting that combinations of parents’ and children’s personal characteristics are associated with child outcomes (Wright & Jackson, 2024). In contrast, the application of RSA to child–parent dyads in adulthood and older age remains limited. Addressing this gap is important as attachment processes and support exchanges continue to play a central role in shaping these relationships across the life course (Mikulincer & Shaver, 2003, 2024). Recent research in the context of romantic relationships has used RSA to demonstrate that congruence and incongruence in partners’ attachment anxiety and avoidance are associated with relational outcomes (Thargay & Giri, 2025). Extending this dyadic approach to adult child–parent relationships, the current study applies RSA to model how combinations of parents’ and adult children’s attachment orientations shape mutual emotional support exchanges, advancing the understanding of how attachment (dis)similarity within the dyad is linked to intergenerational emotional support processes in adulthood and later life.

We developed two dyadic models: one in which emotional support provision by parents and adult children was examined in association with both partners’ levels of attachment anxiety, and the second in association with their levels of attachment avoidance. Each model included two polynomial equations. Applying polynomial regression enabled us to examine how these attachment dimensions in both parents and children shape the levels of emotional support they provide to each other. The analysis considered linear actor effects (*b*1), linear partner effects (*b*2), quadratic actor effects (*b*3), actor-partner interaction effects (*b*4), and quadratic partner effects (*b*5).

Subsequently, we applied response surface analysis to examine how levels of (dis)similarities between adult children’s and their parent’s attachment orientation is associated with their emotional support provision. Response surface analysis visually represents the results of polynomial regression, focusing on the effects of similarity (congruence) and dissimilarity (incongruence) between two predictor variables. The response surface parameters were derived from the polynomial regression coefficients, capturing two slopes and two curvatures along the response surface: (i) The slope along the Line of Congruence (LOC) (*a*1 = *b*1 + *b*2) tests whether emotional support provision increases when parents’ and adult children’s attachment orientation (attachment anxiety or avoidance) align at either higher or lower levels; (ii) The curvature along the LOC (*a*2 = *b*3 + *b*4 + *b*5) examines whether greater similarity in attachment patterns, whether extreme or moderate, corresponds to higher emotional support provision; (iii) The slope along the Line of Incongruence (LOIC) (*a*3 = *b*1 − *b*2) assesses whether differences in attachment anxiety and avoidance between parents and children are associated with increased emotional support provision, and (iv) The curvature along the LOIC (*a*4 = *b*3 − *b*4 + *b*5) evaluates whether emotional support provision increases or decreases based on the degree of similarity or divergence in attachment anxiety and avoidance between parents and children (Hu & Zhou, 2024; Schönbrodt et al., 2018).

We specified two separate dyadic equations for each research model, regressing both parent’s and child’s emotional support on the full set of polynomial terms. Both research models were just-identified models (*CFI* = 1, *TLI* = 1). The equations for our research models were as follows:

Z _parent_ = *b*0 + *b*1 × x (actor effect) _parent_ + *b*2 × y (partner effect) _parent_ + *b*3 × x^2^ (actor effect) _parent_ + *b*4 × x × y (actor effect × partner effect) _parent_ + *b*5 × y^2^ (partner effect) _parent_ + *e*_parent_ (1)

Z _adult child_ = *b*0 + *b*1 × x (actor effect) _adult child_ + *b*2 × y (partner effect) _adult child_ + *b*3 × x^2^(actor effect) _adult child_ + *b*4 × x × y (actor effect × partner’s effect) _adult child_ + *b*5 × y^2^ (partner effect) _adult child_ + *e*_adult child_ (2)

Next, to assess whether parental and child attachment had similar or different effects on their own and their partner’s support provision, we tested whether actor and partner effects differed by role. We applied constraints to both research models, the first examining attachment anxiety and the second examining the attachment avoidance, by equalizing the actor and partner effects. This included equating the linear, quadratic, and interaction effects (*b*1_parent_ = *b*2_adult child_, *b*3_parent_ = *b*5_adult child_; *b*2_parent_ = *b*1_adult child_, *b*5_parent_ = *b*3_adult child_; *b*4_parent_ = *b*4_adult child_). We then compared each constrained model to its respective dyadic research model using *χ*^2^ likelihood ratio test (LRT) (Yang et al., 2024; Schönbrodt et al., 2018). The comparison resulted in *χ*^2^(5) = 14.26, *p* < 0.01 for anxiety model and for the attachment avoidance model it resulted in *χ*^2^(5) = 23.64, *p* < 0.01, indicating that the constrained models provided a poorer fit. This suggests that actor and partner effects on support provision differ between parents and adult children. Based on these results, we analyzed the surfaces, assuming that actor and partner effects differ by role.

### 2.4. Confirmatory Analysis

While RSA represents a complex analytical technique, to our knowledge, no established power analysis method exists specifically for RSA. Nevertheless, it has been applied in previous studies with comparably small samples (Deres-Cohen et al., 2022; Kivlighan et al., 2024). In this study, to validate the findings within our relatively small sample, we followed recommended practices for RSA (Hu & Zhou, 2024; Schönbrodt, 2016). Following these guidelines, to minimize the risk of overfitting and ensure that the results reflect the underlying relationships between variables, we conducted confirmatory analyses by systematically comparing simpler nested models within the full polynomial model using the RSA package in R. In this approach, the software generates models derived from the full polynomial, applying constraints to identify which terms are essential for capturing the relationships in the data. Models within a Δ*AIC* < 2 range are considered statistically equivalent and provide good fit, while absolute performance can be further assessed using descriptive statistics such as adjusted *R*^2^ (*R*^2^adj). This analysis allows evaluation of whether the full polynomial model adequately represents theoretically expected relationships while maintaining parsimony, or whether certain terms add complexity without improving interpretability and can be excluded.

In our analysis, candidate models were initially compared using *AIC* values, with models within Δ*AIC* < 2 considered statistically equivalent. When the full polynomial model did not meet the *AIC* threshold, we assessed constrained models within Δ*AIC* < 2 that retained both actor and partner effects and their interactions, consistent with theoretical expectations. Models containing terms that did not meet these criteria were excluded, while those that met them were considered theoretically supported and served to confirm the model selection. The results of the model selection are presented in the Supplementary Materials.

After identifying the most appropriate model, terms that reduced the interpretability of the results were excluded. In the model examining attachment anxiety, the parent equation retained the full polynomial, whereas the child equation included the linear actor and partner effects (without quadratic terms), and the interaction term. In the model examining attachment avoidance, the parent equations included actor and partner linear effects and their interaction whereas the child equations included only the actor and partner linear effects. Research models were estimated using Maximum Likelihood (ML), with Full Information Maximum Likelihood (FIML) applied to handle missing data. Bootstrapping (*n* = 5000) was applied to compute robust standard errors and *p*-values (Schönbrodt et al., 2018).

## 3. Results

Descriptive statistics and correlations between study variables are presented in Table 1. Parents’ emotional support provision was negatively associated with their own attachment anxiety (*r* = −0.44, *p* < 0.001) and with adult children’s (*r* = −0.32, *p* < 0.001). Similarly, parents’ emotional support provision was negatively associated with their own attachment avoidance (*r* = −0.46, *p* < 0.001) as well as with adult children’s (*r* = −0.39, *p* < 0.001). Adult children’s emotional support provision was negatively associated with their own attachment anxiety (*r* = −0.51, *p* < 0.001) and with their parent’s (*r* = −0.28, *p* < 0.001). Lastly, adult children’s emotional support provision was negatively associated with their own attachment avoidance (*r* = −0.64, *p* < 0.001) and with their parent’s avoidance (*r* = −0.45, *p* < 0.001).

**Figure 1:**
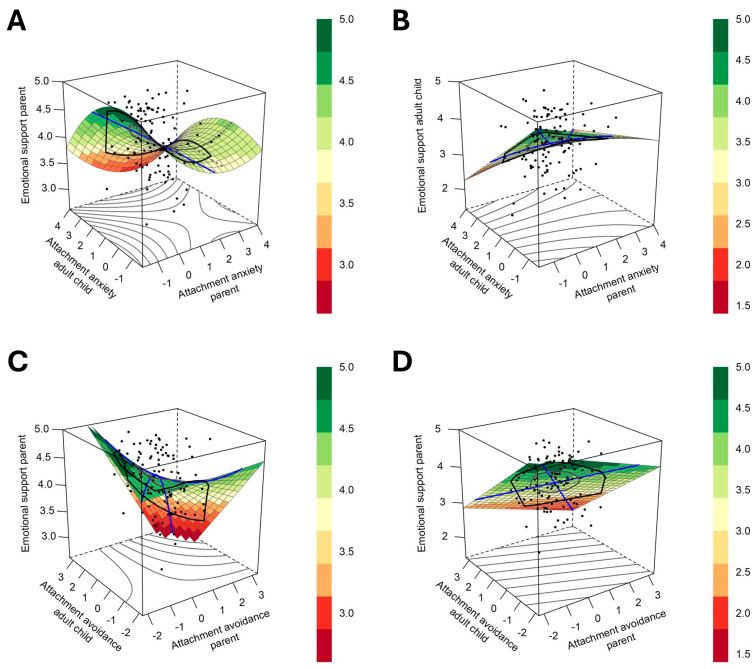
Response surface plots illustrating the associations between emotional support provision and (dis)similarities in parent-child attachment orientations. The X-axis represents the parent’s attachment orientation, the Y-axis represents the adult child’s attachment orientation, and the Z-axis represents emotional support provision. Panels depict attachment anxiety in relation to support provided by parents (**A**) and adult children (**B**), and attachment avoidance in relation to support provided by parents (**C**) and adult children (**D**). Figures were created using R (v.4.3.2) with the RSA package (v.0.10.6) (Schönbrodt & Humberg, 2023).

We examined each research question using Response Surface Analysis (RSA). For each question, we ran a dyadic polynomial regression model and computed the corresponding surface parameters. RQ1 addressed the association between (dis)similarity in attachment anxiety between parents and adult children and the emotional support they provide to one another. RQ2 examined the association between (dis)similarity in attachment avoidance and the emotional support exchanged within the dyad. The results of these analyses are presented in Table 2 and Table 3, respectively. Subsequently, we illustrated the surface findings in Figure 1, corresponding to the analyses of attachment anxiety and avoidance, respectively.

### 3.1. Parents’ and Adult Children’s Attachment Anxiety and Own Emotional Support Provision

The results of the regression coefficients and response surface analysis are presented in Table 2. For parents, following the confirmatory analysis, full polynomial regression was estimated. The RSA findings showed a significant negative slope along the line of congruence (*a*1 = −0.32, *SE* = 0.08, *p* < 0.001), indicating that parental support was higher when both the parent’s and the adult child’s attachment anxiety were aligned at lower levels. Conversely, as similarity in attachment anxiety increased at higher levels, parental support tended to decrease. The curvature along the line of congruence was not significant (*a*2 = 0.03, *SE* = 0.07, *p* = n.s.). This suggests that the effect of similarity in attachment anxiety between parents and adult children on parental support was not explained by whether the matching was at extreme or moderate levels. Along the line of incongruence, a significant negative slope was observed (*a*3 = −0.27, *SE* = 0.10, *p* < 0.01), indicating that parental support increased when the child’s attachment anxiety was higher than the parent’s. However, the curvature along the line of incongruence was not significant (*a*4 = −0.01, *SE* = 0.11, *p* = n.s.). This result indicated that parental support was not explained by a single pattern of matches or mismatches, as matches in anxiety levels between parents and children did not explain higher or lower levels of support provision compared to the mismatches. The results of the response surface analysis are presented in Figure 1 (Panel A).

For adult children, the equation included actor and partner linear effects and the interaction term. The RSA findings showed a significant negative slope along the line of congruence (*a*1 = −0.46, *SE* = 0.08, *p* < 0.001), indicating that adult child’s support was higher when their own and the parent’s attachment anxiety were aligned at lower levels. The non-significant curvature along the line of congruence (*a*2 = 0.06, SE = 0.07, *p* = n.s.) suggests that child’s support was associated with the presence of matching anxiety levels, rather than with their intensity. The slop along the line of incongruence, was significant (*a*3 = −0.26, *SE* = 0.13, *p* < 0.05), indicating that differences in attachment anxiety between the child and the parent were associated with the child’s emotional support. The curvature along the line of incongruence was non-significant (*a*4 = −0.06, *SE* = 0.07, *p* = n.s.), indicating that the similarity in anxiety levels between parents and adult children did not explain significantly higher or lower levels of child’s support provision compared to dissimilarity. See Figure 1 (Panel B).

Although the patterns of support provision were similar between parents and children, the results indicate that their response surfaces differed. For parents, support provision was explained both by linear effects of their own anxiety (*B*1 = −0.30, *SE* = 0.05, *p* < 0.001) and by quadratic effects reflecting higher levels of their own anxiety, which reached marginal significance (*B*3 = 0.07, *SE* = 0.04, *p* = 0.053). In contrast, children’s support for parents with higher anxiety was explained primarily by linear effects of their own anxiety (*B* = −0.36, *SE* = 0.08, *p* = n.s.) when they were at relatively lower levels.

### 3.2. Parent’s and Adult Children’s Attachment Avoidance and Own Emotional Support Provision

The regression coefficients and parameters from the response surface analysis are presented in Table 3. For parents, the equation included both actor and partner linear effects and the interaction term. RSA results indicated significantly negative slope along the line of congruence (*a*1 = −0.35, *SE* = 0.05, *p* < 0.001), showing that parent’s emotional support was higher when both their own and adult child’s attachment avoidance are aligned at lower levels. The curvature along the line of congruence was significant (*a*2 = −0.13, *SE* = 0.06, *p* < 0.05), suggesting that the alignment of attachment avoidance between parents and adult children whether at higher or moderate levels, was significantly linked to the parent’s support provision. Along the line of incongruence, a significant negative slope was observed (*a*3 = −0.20, *SE* = 0.08, *p* < 0.05), showing that parental support increased when the child’s attachment avoidance was higher than the parent’s. The curvature along this line was significantly positive (*a*4 = 0.26, *SE* = 0.15, *p* < 0.05), indicating that higher parental support was associated with greater differences in avoidance levels between parents and children. See Figure 1 (Panel C).

For the adult child, the regression equation included only the actor and partner linear effects. Because the model did not include interaction or quadratic terms (*b*3, *b*5), only the linear slopes (*a*1, *a*3) were estimable. The curvature parameters (*a*2, *a*4) could not be computed, as they are derived from the interaction and quadratic terms. RSA results showed a significantly negative slope along the line of congruence (*a*1 = −0.61, *SE* = 0.06, *p* < 0.001), indicating that adult child’s emotional support was higher when both the parent’s and adult child’s attachment avoidance were aligned at lower levels. The slope along the line of incongruence was significantly positive (*a*3 = −0.27, *SE* = 0.11, *p* < 0.01), showing that the adult child’s support increased when the parent’s attachment avoidance is higher than their own. See Figure 1 (Panel D).

The response surfaces revealed a similar pattern for both parents and children: support increased when the partner’s avoidance was higher than the actor’s and when both reported low levels of avoidance. However, the model comparisons conducted prior to the response surface analysis indicated that actor and partner effects differ between parents and children, leading us to examine how avoidance operates differently within each role. The results showed that the significant regression coefficients shaping the surface for parents were the linear actor effects (*b*1 = −0.28, *SE* = 0.05, *p* < 0.001) and the interaction between their own and their child’s avoidance (*b*4 = −0.13, *SE* = 0.06, *p* < 0.05). In contrast, the child’s surface was shaped by significant linear actor and partner effects (*b*1 = −0.17, *SE* = 0.06, *p* < 0.01; *b*2 = −0.44, *SE* = 0.07, *p* < 0.001). These results suggest that although the overall patterns of support provision were similar between parents and adult children, the parents’ surface was driven by the interaction between their own and their child’s avoidance, whereas the children’s surface was driven by the linear actor and partner effects.

## 4. Discussion

This study aimed to provide an attachment theory perspective to investigate emotional support between older parents and adult children. We examined how similarities and differences in adult child–parent attachment-related anxiety and avoidance explain the emotional support they provide to each other. Given that attachment-related anxiety and avoidance have different implications for relationship functioning and support patterns (e.g., Collins & Feeney, 2000; Mikulincer & Shaver, 2023a), we analyzed these attachment dimensions separately in two dyadic research models.

### 4.1. (Dis)Similarities in Parents’ and Adult Children’s Attachment Anxiety and Their Own Emotional Support Provision

The first research question RQ1 examined how the (dis)similarities in levels of anxiety between parents and adult children are associated with the emotional support they provide to each other. The results indicate that parent’s emotional support provision is significantly shaped by their own attachment anxiety, while also reflecting the anxiety levels of the child. We found that parents offer greater emotional support to their children when they both share lower levels of anxiety, compared to when they experience similarly high anxiety levels. These findings align with previous studies suggesting that attachment anxiety is linked to an individual’s self-focused orientation toward seeking reassurance and attention from the relational partner, limiting their mental availability to address the partner’s emotional support needs (Mikulincer & Shaver, 2003, 2023b).

The current research’s findings also indicate that while parental anxiety can hinder emotional responsiveness when parents experience higher anxiety than the child’s, parents provide greater emotional support when the child’s anxiety exceeds their own. Parents provided higher emotional support when child’s anxiety was higher than their own, even when the parents themselves reported high levels of anxiety. Previous studies have shown that the persistent demands and signs of distrust often seen in individuals with high anxiety can place an emotional strain on their partner’s support efforts, leading to growing distance, and potential rejection rather than emotional connection (Mikulincer & Shaver, 2003, 2020, 2023b). However, in adult child–parent dyads, parents may be guided by their lifelong caregiving role, which enables them to provide support even when experiencing their own insecurity and vulnerability, helping them navigate relationship tensions and remain emotionally responsive to children with high attachment anxiety.

Similarly to parents, adult children provided greater support when both they and their parents reported lower attachment anxiety. However, they offered more support to highly anxious parents only when their own anxiety was low and struggled to meet parental emotional needs as their own anxiety increased. This finding can be examined from a developmental perspective, as research suggests that developmental stages from early to middle adulthood are often marked by the formation of social roles outside the family of origin and the establishment of independence and personal growth (Buchinger et al., 2022; Ebner et al., 2006). Therefore, the child’s motivation to provide support, driven by their own anxiety independent of the parent’s, may reflect children’s difficulty in responding to parental anxiety cues as these may signal vulnerability and dependency, potentially posing a threat of role reversal and conflict with the child’s primary developmental goals. Furthermore, the current study suggests that adult children may be more burdened by their own anxiety, limiting their ability to respond to their parents’ emotional needs and creating a barrier to the reciprocal exchange of emotional support with aging parents. These findings align with prior research indicating that for children with high attachment anxiety, parental signs of vulnerability may confront them with self-focused concerns and doubts about whether they deserve a supportive relationship in which their own needs can be met, which may limit their responsiveness and provision of emotional care (Carpenter, 2001; Mikulincer & Shaver, 2020, 2024).

### 4.2. (Dis)Similarities in Parents’ and Adult Children’s Attachment Avoidance and Their Own Emotional Support Provision

The second research questions RQ2 investigated how the (dis)similarities in avoidance levels between parents and adult children are associated with the emotional support they provide to each other. In line with previous findings showing that high avoidance is associated with withdrawal from a partner’s interpersonal needs (Collins & Feeney, 2000; Mikulincer & Shaver, 2023b), the results indicate that lower avoidance allows both parents and adult children to offer greater emotional support, whereas higher avoidance limits their ability to maintain emotionally supportive connection.

In contrast to previous findings suggesting that individuals experience growing frustration and emotional distance over time when their partner is highly avoidant and dismissive of support attempts (Mikulincer & Shaver, 2003, 2023b), we found that parents and adult children provide greater support when the partner’s avoidance is higher than their own. Although the findings revealed a similar pattern for both parents and children, the underlying processes driving support varied based on the role.

Parents’ support provision was shaped by both their own level of avoidance and its interaction with their child’s avoidance. This suggests that parents may regulate their caregiving responses toward a more avoidant child by integrating the child’s emotional distancing into their supportive behavior. This regulation may reflect the emotional significance parents ascribe to the relationship with their children (Birditt et al., 2015; Zhou et al., 2024), possibly activating caregiving efforts aimed at compensating for emotional distance in an effort to optimize and maintain the relationships as a mutual source of emotional support and connection (Collins & Ford, 2010; Gillath et al., 2005; Mikulincer & Shaver, 2023b, 2024). On the other hand, adult children rely on their own attachment avoidance when interpreting the parent’s avoidance and providing them support. This suggests that although children provide more emotional support to a highly avoidant parent, their response may reflect a personal need to maintain connection, shaped by their internal attachment lens, with limited ability to regulate their own caregiving responses to the avoidant parent’s emotional cues when offering them support. These role-based asymmetries can provide a broader perspective on the mechanisms through which parents and adult children navigate and respond to relational distance and preserve the emotional connection.

The observed generational asymmetries in this study between parents and adult children can be further understood through life-course and intergenerational role perspectives. For parents, providing support to their children is considered a lifelong role, encompassing emotional attunement and responsiveness to the child’s needs (Fingerman et al., 2024). Adult children, by contrast, are more engaged with their own developmental transitions, such as leaving the parental home, establishing their own families, while navigating parental aging, which can shape the opportunities to meet their parents’ emotional needs and their capacity to reciprocate support as research shows that parents remain more invested in providing support to their children than children are toward their parents (Birditt et al., 2015; Bui et al., 2022). Interpersonal processes of emotional support, guided by attachment, may interact with these normative developmental trajectories, shaping how adult child–parent dyads navigate emotional support exchanges.

### 4.3. Limitations and Further Research

This study presents important strengths including the reliance on dyadic data, but several limitations should also be noted. First, the present study employed a cross-sectional design, which limits the ability to draw causal conclusions. Examining emotional support exchanges between parents and children across time and different life stages can deepen the understanding of the role of attachment in maintaining emotional connectedness amid the gradual decline and transformation of support systems with age (Carstensen et al., 2003; Carstensen, 2021). Future research should employ a longitudinal design to examine the temporal associations between attachment function and emotional support provision in adult child–parent dyads. Second, although the study is based on a relatively small sample, which may limit the generalizability of the findings, it employs an analysis that validates the results and provides a method for examining the complexity of parent–child relationships. This approach offers a dyadic perspective that expands theoretical understanding and contributes to current knowledge of attachment’s role in intergenerational relationships across adulthood and older age, warranting further investigation in larger cohorts. Moreover, intergenerational support norms and attachment-related expectations are embedded within cultural frameworks that shape reciprocity, affective solidarity, and role expectations in adult child–parent relationships (Garcia-Mendoza et al., 2024). The present study was conducted in Israel, a highly family-oriented society, where strong norms of familial support may promote close and supportive connections between parents and adult children (Bijaoui & Katz, 2024). Examining attachment-related support exchanges in more diverse cultural contexts may therefore enhance the generalizability of findings and clarify how attachment processes operate within families across different sociocultural settings. Third, the study relied on self-report measures, which may limit the understanding of how support and emotional connection manifest in interpersonal interactions. As individuals perceive their own and relational partner’s behaviors through the lens of their own attachment (Mikulincer & Shaver, 2023a), discrepancies may arise between self-reported and observed or perceived behavior. Future research should incorporate observational methods together with self-report to provide a more comprehensive perspective on the role of attachment and support behaviors in adult child–parent relationships. Furthermore, the examination of attachment anxiety and avoidance in separate research models limits our ability to draw conclusions about the effects of attachment security or other specific interactions of attachment dimensions. Further research could shed light on how attachment functions within intergenerational support by examining interaction patterns between anxiety and avoidance and exploring how they are associated with support behaviors and emotional connection. As anxiety and avoidance reflect distinct regulatory strategies, cross-dimensional combinations could reveal additional ways in which attachment shapes emotional support and relational functioning, beyond the within-dimension effects (Mikulincer & Shaver, 2023a, 2024). Finally, In the present study, participants were allowed to select which parent or child would participate with them to ensure their comfort. However, it is important to acknowledge that parents and children may have different emotional connections with different family members (Fingerman et al., 2020, 2024), which could shape attachment-based processes and patterns of emotional support. Future research focusing on the emotional and attachment aspects of child–parent relationships should examine multiple child–parent dyads within the same family to better understand variation in support dynamics. Additionally, although our sample included all gender combinations of adult child–parent dyads (i.e., mother-daughter, father-daughter, mother-son, father-son), these categories were somewhat unevenly represented. Given that women and men may be socialized differently with respect to emotional expression (Lewis & Michalson, 2025), and the role of gender in shaping child–parent relational support (Ayalon & Segel-Karpas, 2022; Segel-Karpas & Ayalon, 2022), future studies should aim for a more balanced representation of same-gender and different-gender dyads to explore how gender may shape attachment-based interactions, patterns of emotional support, and in adult child–parent relationships.

Despite its limitations, this study offers theoretical and practical implications. Theoretically, the study offers a dyadic psychological perspective on the internal mechanisms and interpersonal processes shaping emotional support between parents and their adult children. By employing a dyadic perspective, the study illustrates how similarities and differences in attachment orientation shape each generation’s motivation to maintain emotional connectedness, which varies between adult children and aging parents. The study provides evidence for reciprocal dynamics in child–parent relationships, showing how intergeneration support is based on emotional needs and attachment cues. In that, this study paves the way for future research to explore how attachment contributes to social motivation in later life, enabling individuals to address relational emotional needs, preserve the continuity of close relationships and their social identity, while navigating loss and aging. Practically, these findings can inform therapeutic and psychoeducational efforts by guiding the development of attachment-based interventions for adult child–parent dyads. By identifying patterns of attachment similarity, practitioners can tailor attachment-based interventions, family counselling, and psychosocial support programs to the dyad’s specific relational needs. This allows them to target concrete processes, such as effective communication skills, teaching emotion-regulation strategies, or supporting reciprocal responsiveness, promoting more effective emotional support exchanges. Focusing on attachment similarity also allows interventions to build on existing relational strengths, contributing to mutually supportive adult child–parent interactions across adulthood and older age, and supporting relational continuity and psychological well-being in later life.

## 5. Conclusions

The present study examined how (dis)similarity in parents’ and adult children’s attachment orientations are associated with the emotional support exchanged within the dyad. The first research question focused on the association between parents’ and adult children’s attachment anxiety and their own emotional support provision. The findings showed an asymmetric caregiving patterns between parents and adult children. Parents were able to provide higher levels of emotional support when their child’s attachment anxiety exceeded their own, including at higher levels of child anxiety. In contrast, adult children’s emotional support provision was more constrained when parental attachment anxiety was at higher levels. This pattern suggests that parents may continue in their caregiving role in adulthood by remaining responsive to elevated child distress, whereas adult children may experience greater emotional burden when confronted with heightened parental anxiety, which may limit their capacity to reciprocate emotional support.

The second research question examined how parents’ and adult children’s attachment avoidance was associated with their own emotional support provision. Results showed a similar pattern, where both parents and adult children provided higher levels of emotional support when their partner’s avoidance exceeded their own. However, the underlying interpersonal processes was different between dyad members. Parents’ support provision was shaped by both their own level of attachment avoidance and its interaction with their child’s avoidance, suggesting an adaptive regulation of caregiving in which parents may incorporate the child’s emotional distancing into their supportive responses. In contrast, adult children’s responses were driven by their own attachment avoidance when interpreting and responding to parental avoidance, suggesting that children’s support may reflect a personal motivation to maintain connection rather than adjustment to the parent’s emotional cues.

These findings highlight asymmetry in attachment-based caregiving processes within adult child–parent relationships in adulthood and later life, consistent with the family development perspective and prior research showing that even as support exchanges may become more mutual within child–parent bond in later life, parental caregiving remains a salient role across the life course as parents navigate aging (Carter & McGoldrick, 2005; Fingerman et al., 2024). While parents are capable of maintaining caregiving responses to their children’s heightened attachment needs, adult children may be more constrained by attachment-related insecurities, which may limit their ability to reciprocate emotional support. Future research should examine these processes in larger and more diverse samples and across cultural contexts to further clarify how attachment dynamics shape intergenerational support in later life.

## Figures and Tables

**Table 1:** Descriptive statistics and correlations between study variables.

	M	SD	1	2	3	4	5
1. Emotional support parent	4.33	0.58					
2. Emotional support adult child	3.93	0.75	0.43 ***				
3. Attachment anxiety parent	2.34	0.88	−0.44 ***	−0.28 **			
4. Attachment avoidance parent	3.00	0.80	−0.46 ***	−0.45 ***	0.61 ***		
5. Attachment anxiety adult child	2.59	1.08	−0.32 ***	−0.51 ***	0.32 **	0.25 **	
6. Attachment avoidance adult child	3.15	1.12	−0.39 ***	−0.64 ***	0.41 ***	0.34 ***	0.72 ***

Note: ** *p* < 0.01, *** *p* < 0.001.

**Table 2:** Parents’ and adult children’s emotional support provision and attachment anxiety.

	Emotional Support Provision Parents			Emotional Support Provision Adult Children		
Regression coefficients	B	SE	95% CI	B	SE	95% CI
Actor effects linear (*b*1)	−0.30 ***	0.05	[−0.41, −0.20]	−0.36 ***	0.08	[−0.53, −0.21]
Partner effects linear (*b*2)	−0.03	0.07	[−0.17, 0.10]	−0.10	0.07	[−0.24, 0.03]
Actor effects squared (*b*3)	0.07 +	0.04	[0.01, 0.16]			
Actor × partner (*b*4)	0.02	0.06	[−0.13, 0.12]	0.05	0.07	[−0.10, 0.20]
Partner effects squared (*b*5)	−0.06	0.05	[−0.14, 0.04]			
**Response surface parameters**						
Slope along LOC (*a*1)	−0.32 ***	0.08	[−0.49, −0.19]	−0.46 ***	0.08	[−0.62, −0.31]
Curvature along LOC (*a*2)	0.03	0.07	[−0.14, 0.15]	0.06	0.07	[−0.10, 0.20]
Slope along LOIC (*a*3)	−0.27 **	0.10	[−0.46, −0.08]	−0.26 *	0.13	[−0.52, −0.02]
Curvature along LOIC (*a*4)	−0.01	0.11	[−0.19, 0.24]	−0.06	0.07	[−0.20, 0.10]

Note: LOC refers to the Line of Congruence, and LOIC refers to the Line of Incongruence in the response surface analysis. For parents, the regression equation included the full polynomial, while for children it included both linear actor and partner effects and the interaction term. The empty cells in the table are not included in the calculations because of the model constraints. * *p* < 0.05, ** *p* < 0.01, *** *p* < 0.001, + *p* < 0.10.

**Table 3:** Parents’ and adult children’s emotional support provision and attachment avoidance.

	Emotional Support Provision Parents			Emotional Support Provision Adult Children		
Regression coefficients	B	SE	95% CI	B	SE	95% CI
Actor effects linear (*b*1)	−0.28 ***	0.05	[−0.38, −0.19]	−0.44 ***	0.07	[−0.58, −0.32]
Partner effects linear (*b*2)	−0.07	0.05	[−0.18, 0.03]	−0.17 **	0.06	[−0.28, −0.06]
Actor effects squared (*b*3)						
Actor × partner (*b*4)	−0.13 *	0.06	[−0.26, −0.01]			
Partner effects squared (*b*5)						
**Response surface parameters**						
Slope along LOC (*a*1)	−0.35 ***	0.05	[−0.46, −0.25]	−0.61 ***	0.06	[−0.74, −0.50]
Curvature along LOC (*a*2)	−0.13 *	0.06	[−0.26, 0.01]			
Slope along LOIC (*a*3)	−0.20 *	0.08	[−0.37, −0.04]	−0.27 **	0.11	[−0.48, −0.07]
Curvature along LOIC (*a*4)	0.14 *	0.06	[0.01, 0.26]			

Note: LOC refers to the Line of Congruence, and LOIC refers to the Line of Incongruence in the response surface analysis. For parents, the regression equation included both linear actor and partner effects and the interaction term, while for children it included the linear actor and partner effects. The empty cells in the table are not included in the calculations because of the model constraints. * *p* < 0.05, ** *p* < 0.01, *** *p* < 0.001.

## Data Availability

The data that support the findings of this study are available from the corresponding author upon reasonable request.

## Supplementary Materials

The following supporting information can be downloaded at: https://www.mdpi.com/article/10.3390/bs16010106/s1, Table S1: Parental and adult child attachment anxiety and parental emotional support provision; Table S2: Parental and adult child attachment anxiety and adult child‘s emotional support provision; Table S3: Parental and adult child attachment avoidance and parental emotional support provision; Table S4: Parental and adult child attachment avoidance and adult child‘s emotional support provision.
