# Supplementary material for: The Role of Attachment in Emotional Support Provision in Adult Child–Parent Relationships: A Dyadic Response Surface Analysis
Source: Behav Sci (Basel). 2026 Jan 13;16(1):106. doi: 10.3390/bs16010106 (PMC12837992; doi:10.3390/bs16010106)
Supplement: Supplementary file 1 [file behavsci-16-00106-s001.zip › behavsci-4028052-supplementary.pdf]

Supplemental Material

for

The Role of Attachment in Emotional Support Provision in Adult Child-Parent Relationships: A Dyadic Response Surface Analysis

Supplemental Material for the confirmatory analyses: Model Selection

Table S1

Parental and adult child attachment anxiety and parental emotional support provision

Model Selection	k	AICc	ΔAICc	CFI	R^2_{adj}	$_p$model
SRSQD	5	159.33	.00	.97	.240	<.001
Only actor effects, quadratic	4	160.80	1.47	.88	.221	<.001
**Full Polynomial**	**7**	**161.29**	**1.96**	**1.00**	**.244**	**<.001**
SRRR	6	161.58	2.25	.94	.233	<.001
Additive	4	161.78	2.45	.85	.214	<.001
RR	4	163.25	3.91	.80	.202	<.001
IA	5	163.44	4.11	.84	.210	<.001
SRR	5	163.98	4.64	.82	.206	<.001
Only actor effects, linear	3	164.11	4.78	.73	.187	<.001
Only partner effects, linear	3	175.17	16.17	.34	.093	<.001
Only partner effects, quadratic	4	177.36	18.03	.32	.086	<.01
SQD	3	182.24	22.90	.12	.032	<.05
SSQD	4	182.24	22.91	.16	.042	<.05
Null	2	184.53	25.20	.00	NA	<.001

Note: Selected model is presented in Bold; K, number of parameters in the model; AICc, corrected Akaike Information Criterion; CFI, Comparative fit index; R^2_{adj}, adjusted R, represents the proportion of variance in the dependent variable explained by the model, adjusted for predictors and sample size; $_p$model, p-value for the model's explained variance; Model abbreviations: SRSQD, Shifted and Rotated Squared Differences model; SRRR, Shifted and Rotated Rising Ridge Model; Additive, a model that includes both parent's (x) and adult child's linear effects (y); RR, Rising Ridge Model; IA, Moderated regression, a model that includes both parent's (x) and adult child's linear effects (y), and the interaction term (x*y); SRR, Shifted Rising Ridge Model; SQD, Basic Squared Difference Model; SSQD, Shifted Squared Difference Model; Null, null model, includes only the intercept.

Table S2

Parental and adult child attachment anxiety and adult child‘s emotional support provision

Model Selection	k	AICc	ΔAICc	CFI	R^2_{adj}	$_{p}$model
Only actor effects, linear	3	210.88	.00	1.00	.258	<.001
Additive	4	211.17	.29	1.00	.265	<.001
Only actor effects, quadratic	4	212.70	1.82	1.00	.254	<.001
**IA**	**5**	**212.80**	**1.92**	**1.00**	**.261**	**<.001**
SRSQD	5	213.11	2.23	1.00	.259	<.001
SRR	5	213.37	2.49	1.00	.257	<.001
SRRR	6	214.98	4.10	1.00	.254	<.001
RR	4	216.88	6.00	.89	.223	<.001
Full polynomial	7	217.25	6.37	1.00	.247	<.001
Only partner effects, linear	3	234.70	23.82	.25	.068	<.01
SSQD	4	234.87	23.99	.28	.076	<.01
SQD	3	235.83	24.95	.21	.057	<.01
Only partner effects, quadratic	4	235.87	24.99	.25	.067	<.05
Null	2	240.88	30.00	.00	.00	N.A

Note: Selected model is presented in Bold; K, number of parameters in the model; AICc, corrected Akaike Information Criterion; CFI, Comparative fit index; R^2_{adj}, adjusted R, represents the proportion of variance in the dependent variable explained by the model, adjusted for predictors and sample size; $_{p}$model, p-value for the model's explained variance; Model abbreviations: Additive, a model that includes both parent's (x) and adult child's linear effects (y); IA, Moderated regression, a model that includes both parent's (x) and adult child's linear effect (y), and the interaction term (x*y); SRSQD, Shifted and Rotated Squared Differences model; SRR, Shifted Rising Ridge Model; SRRR, Shifted and Rotated Rising Ridge Model; RR, Rising Ridge Model; SSQD, Shifted Squared Difference Model; SQD, Basic Squared Difference Model; Null, null model, includes only the intercept.

Table S3

Parental and adult child attachment avoidance and parental emotional support provision

Model Selection	k	AICc	ΔAICc	CFI	R^2_{adj}	$_p$model
**IA**	**5**	**144.86**	**.00**	**1.00**	**.301**	**<.001**
SRR	5	147.44	2.58	.95	.283	<.001
Additive	4	147.54	2.68	.92	.274	<.001
SRSQD	5	148.25	3.39	.93	.278	<.001
Full polynomial	7	148.33	3.47	1.00	.294	<.001
Only actor effects, linear	3	148.68	3.82	.85	.258	<.001
RR	4	148.97	4.11	.88	.264	<.001
SRRR	6	149.69	4.83	.93	.276	<.001
Only actor effects, quadratic	4	150.75	5.89	.83	.251	<.001
Only partner effects, linear	3	166.87	22.01	.35	.115	<.001
Only partner effects, quadratic	4	169.02	24.16	.32	.106	<.01
Null	2	178.32	33.46	.00	.00	N.A
SSQD	4	180.03	35.17	.02	.005	N.S
SQD	3	180.44	35.58	.00	.00	N.S

Note: Selected model is presented in Bold; K, number of parameters in the model; AICc, corrected Akaike Information Criterion; CFI, Comparative fit index; R^2_{adj}, adjusted R, represents the proportion of variance in the dependent variable explained by the model, adjusted for predictors and sample size; $_p$model, p-value for the model's explained variance; Model abbreviations: IA, Moderated regression, a model that includes both parent's (x) and adult child's linear effects (y), and the interaction term (x*y); SRR, Shifted Rising Ridge Model; Additive, a model that includes both parent's (x) and adult child's linear effects (y); SRSQD, Shifted and Rotated Squared Differences model; RR, Rising Ridge Model; SRRR, Shifted and Rotated Rising Ridge Model; Null, null model, includes only the intercept; SSQD, Shifted Squared Difference Model; SQD, Basic Squared Difference Model.

Table S4

Parental and adult child attachment avoidance and adult child's emotional support provision

Model Selection	k	AICc	ΔAICc	CFI	R^2_{adj}	$_p$model
**Additive**	**4**	**176.69**	**.00**	**1.00**	**.469**	**<.001**
SRSQD	5	177.99	1.30	1.00	.468	<.001
SRR	5	178.62	1.93	1.00	.465	<.001
IA	5	178.81	2.12	1.00	.464	<.001
SRRR	6	180.24	3.55	1.00	.463	<.001
Only actor effects, linear	3	182.46	5.77	.92	.432	<.001
Full polynomial	7	182.53	5.84	1.00	.457	<.001
RR	4	183.49	6.80	.92	.432	<.001
Only actor effects, quadratic	4	184.55	7.86	.91	.426	<.001
Only partner effects, linear	3	217.34	40.65	.37	.203	<.001
Only partner effects, quadratic	4	219.43	42.74	.36	.195	<.001
SSQD	4	237.20	60.51	.07	.044	<.05
SQD	3	239.18	62.49	.02	.014	N.S
Null	2	239.57	62.88	.00	.00	N.A

Note: Selected model is presented in Bold; K, number of parameters in the model; AICc, corrected Akaike Information Criterion; CFI, Comparative fit index; R^2_{adj}, adjusted R, represents the proportion of variance in the dependent variable explained by the model, adjusted for predictors and sample size; $_p$model, p-value for the model's explained variance; Model abbreviations: Additive, a model that includes both parent's (x) and adult child's linear effects (y); SRSQD, Shifted and Rotated Squared Differences model; SRR, Shifted Rising Ridge Model; IA, Moderated regression, a model that includes both parent's (x) and adult child's linear effects (y), and the interaction term (x*y); SRRR, Shifted and Rotated Rising Ridge Model; RR, Rising Ridge Model; SSQD, Shifted Squared Difference Model; SQD, Basic Squared Difference Model; Null, null model, includes only the intercept.
